# Resection Followed by Involved-Field Fractionated Radiotherapy in the Management of Single Brain Metastasis

**DOI:** 10.3389/fonc.2015.00206

**Published:** 2015-09-22

**Authors:** Samuel M. Shin, Ralph E. Vatner, Moses Tam, John G. Golfinos, Ashwatha Narayana, Douglas Kondziolka, Joshua Seth Silverman

**Affiliations:** ^1^Department of Radiation Oncology, New York University Langone Medical Center, New York, NY, USA; ^2^Department of Neurosurgery, New York University Langone Medical Center, New York, NY, USA; ^3^Department of Radiation Oncology, Greenwich Hospital, Greenwich, CT, USA

**Keywords:** involved-field fractionated radiotherapy, stereotactic radiosurgery, brain metastases, surgical resection

## Abstract

**Introduction:**

We expanded upon our previous experience using involved-field fractionated radiotherapy (IFRT) as an alternative to whole brain radiotherapy or stereotactic radiosurgery for patients with surgically resected brain metastases (BM).

**Materials and methods:**

All patients with single BM who underwent surgical resection followed by IFRT at our institution from 2006 to 2013 were evaluated. Local recurrence (LR)-free survival, distant failure (DF)-free survival, and overall survival (OS) were determined. Analyses were performed associating clinical variables with LR and DF. Salvage approaches and toxicity of treatment for each patient were also assessed.

**Results:**

Median follow-up was 19.1 months. Fifty-six patients were treated with a median dose of 40.05 Gy/15 fractions with IFRT to the resection cavity. LR-free survival was 91.4%, DF-free survival was 68.4%, and OS was 77.7% at 12 months. No variables were associated with increased LR; however, melanoma histopathology and infratentorial location were associated with DF on multivariate analysis. LRs were salvaged in 5/8 patients, and DFs were salvaged in 24/29 patients. Two patients developed radionecrosis.

**Conclusion:**

Adjuvant IFRT is feasible and safe for well-selected patients with surgically resected single BM. Acceptable rates of local control and salvage of distal intracranial recurrences continue to be achieved with continued follow-up.

## Introduction

Brain metastases (BM) are the most common central nervous system (CNS) tumors in adults (1, 2). Prognosis is poor according to a recursive partitioning analysis (RPA) estimating a median survival of 2.3–7.1 months for patients treated with whole brain radiotherapy (WBRT) (3). Some patients live longer, as demonstrated by a graded prognostic assessment based on tumor pathology, estimating median survival as high as 18.7 months for patients with limited risk factors (4). A nomogram was developed to provide individualized estimates of survival (5). In the breast cancer population, a recently validated modified breast graded prognostic assessment demonstrated improvement in overall survival (OS) in patients with ≤3 BM compared to >3 BM. The best estimated median OS in patients with favorable risk factors using this index was as high as 28.8 months (6). Despite these tools, it is still difficult to accurately predict the survival of individual patients with BM as some patients survive years after treatment (7). Improvements in systemic therapy and understanding of tumor biology may further improve the outlook of this population, with an increasing emphasis on enhancing quality of life after treatment (8, 9).

Therapeutic options for BM include corticosteroids, surgery, WBRT, and stereotactic radiosurgery (SRS). Two phase three randomized trials demonstrated decreased LR and improved OS and quality of life for patients with single BM treated with surgery combined with WBRT compared to WBRT alone (10, 11). The importance of WBRT following surgery was demonstrated in a separate randomized trial, showing that WBRT decreases the risk of LR, DF, and neurologic death, with no improvement in OS or functional independence (12).

These outcomes justify use of WBRT following surgical resection of single BM; however, these benefits come at the cost of late neurocognitive side effects. Due to improvements in systemic therapy, patients can live to experience the late effects of WBRT, prompting an interest in improving control of intracranial disease while minimizing late neurocognitive effects (13–15). Treatment with memantine and hippocampal-sparing radiotherapy techniques are some strategies used to decrease neurocognitive decline following WBRT (16, 17).

Since recurrences often occur at the surgical cavity following metastasectomy, another approach is to treat the surgical cavity with adjuvant radiotherapy to decrease LR while sparing the majority of the brain. Focal approaches including SRS have been used to improve control of intracranial disease, while decreasing late neurocognitive toxicity following surgical resection of BM (18–22). Another approach is to use involved-field fractionated radiotherapy (IFRT). The purpose of this study is to expand upon our reported experience and present the long-term outcomes including LR, DF, and OS using IFRT in this population. We also analyzed variables associated with LR and DF in this patient population.

## Materials and Methods

From 2006 to 2013, 56 consecutive patients with single BM underwent gadolinium-enhanced pre-operative magnetic resonance imaging (MRI) or computed tomography (CT) of the brain if MRI was contraindicated followed by surgical removal of BM followed by IFRT. Patients who were selected for this treatment approach had good performance status with either controlled primary or extracranial metastatic disease. In addition to good performance status and well-controlled metastatic disease, patients were selected for this treatment approach to improve pre-treatment neurological symptoms, relieve mass effect, or provide histopathological confirmation of metastatic disease. Patients underwent post-operative MRI within 48 h to confirm extent of surgery. This study was approved by our institutional review board.

## Radiotherapy Planning and Treatment

Prior to radiotherapy, CT simulation was performed. Patients were immobilized using a thermoplastic head mask, and 1.25 mm axial CT images were obtained using a LightSpeed RT 16 CT Simulator (GE Healthcare, Waukesha, WI, USA). The images were transferred to the Eclipse treatment planning system (Varian, Palo Alto, CA, USA) and registered with the pre-operative T1-weighted pre and post-contrast and T2-weighted MRI to aid in target delineation. In patients who were unable to undergo MRI examination due to contraindications (i.e., presence of a cardiac pacemaker), the CT simulation images were registered with the pre-operative pre- and post-contrast CT of the brain.

The surgical resection cavity was identified on planning CT, and was defined as the clinical target volume (CTV). Substantial collapse of the surgical cavity identified on post-operative MRI or CT brain was treated with SRS. The planning target volume (PTV) was defined as a 10 mm isometric margin around the CTV (23). Radiotherapy was planned to a dose of 30–40.05 Gy in 10–15 fractions prescribed so that 100% of the PTV received 95% of the dose. The rationale for using this dose is based on its biological equivalence to a dose of 36 Gy/12 fractions used in a study conducted by Patchell et al., assuming an α/β ratio of 2 for CNS tissue and 10 for brain tumor (10). Treatment was delivered using a Varian Clinac 21EX or 2100c linear accelerator (Varian, Palo Alto, CA, USA) with three-dimensional conformal or intensity-modulated radiotherapy using 4 MV/6 MV photons.

## Follow-Up

Patients had clinical and radiologic follow-up with neurological examination and MRI scans 1 month after completion of IFRT and every 3 months thereafter.

## Data Collection

All patients with single BM who underwent surgery followed by IFRT from 2006 to 2013 were identified by reviewing the clinical charts of patients treated in our department, with patient characteristics collected and recorded in a database.

## Statistical Analysis

Clinical endpoints include LR, defined as the presence of new enhancement along the resection cavity identified on serial MRI, and DF, defined as enhancement consistent with BM and/or leptomeningeal spread outside the IFRT volume. Time to local or distant progression was defined from the date of surgery to the first MRI demonstrating recurrence. OS was calculated from date of BM diagnosis until date of death. Parameters associated with LR and/or DF were assessed including active extracranial disease, histopathology of primary tumor [non-small cell lung cancer (NSCLC), breast, or melanoma], tumor maximal diameter (<3 cm or ≥3 cm), *en bloc* versus piecemeal surgical resection, lesion location (infratentorial versus supratentorial), and tumor depth (superficial with dural involvement versus deep parenchymal invasion). Statistical analysis was performed using SPSS software (version 20.0, IBM, Armonk, NY, USA). The Kaplan–Meier method was used to calculate the rate of OS, LR-free survival, and DF-free survival. Univariate and multivariate analyses of significance were performed using Cox regression analysis. A *p*-value of 0.05 or less was considered statistically significant.

## Results

Fifty-six patients with single BM who underwent surgery followed by IFRT were included in this study. Detailed characteristics of the study group are shown in Table 1. Forty-seven (84%) presented with neurological symptoms, and seven presented without neurological symptoms identified incidentally on brain imaging.

**Table 1:** **Patient characteristics**.

Characteristics	*n* = 56	%
**Gender**
Men	19	33.9
Women	37	66.1
**Age (years)**
Median (range)	58 (range: 27–82)	
**RPA class**
I	21	37.5
II	34	60.7
III	1	1.8
**KPS score**
60	1	1.8
70	4	7.1
80	16	28.6
90	35	62.5
Median (range)	90 (range: 60–90)	
**Extracranial metastases**
Present	28	50.0
Absent	28	50.0
**Primary cancer**
NSCLC	20	35.6
Breast	15	26.8
Melanoma	11	19.6
Unknown	3	5.4
Ovary	3	5.4
Gastric	1	1.8
Bladder	1	1.8
Sarcoma	1	1.8
Endometrium	1	1.8
**Tumor size (cm)**
Mean (range)	3.4 (range: 1.7–5.7)	
**Tumor location**
Cerebellum	17	30.3
Frontal	11	19.6
Parietal	10	17.9
Temporal	9	16.1
Occipital	8	14.3
Thalamus	1	1.8

## Surgical Treatment Characteristics

All 56 patients underwent gross total resection (GTR), with 27 (48%) having their BM removed *en bloc* and 29 (52%) with piecemeal resection. The most common tumor histopathology was NSCLC in 20 (36%), breast cancer in 15 (27%), and melanoma in 11 patients (20%). Thirty-eight (68%) had supratentorial lesions, while 18 (32%) had infratentorial lesions. Seventeen (30%) had superficial lesions (involvement/abutment of dura) and 39 (70%) had deep lesions (surrounded by parenchyma). Mean tumor size (tumor maximal dimension determined by MRI) was 3.4 cm (range, 1.7–6 cm), with 35/56 having tumors ≥3 cm in size.

## Radiotherapy Characteristics

**Figure 1:**
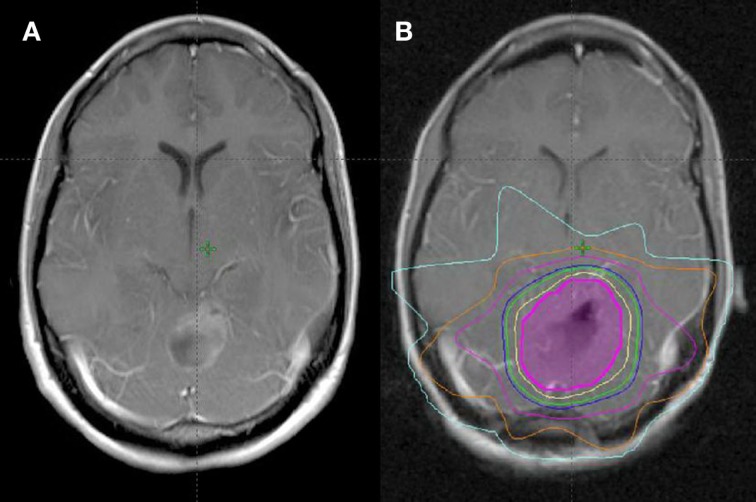
**Treatment planning following GTR of single BM**. **(A)** Pre-operative MRI (T1 post). **(B)** Post-operative MRI fused with planning CT for treatment planning (magenta = CTV, yellow = 100% isodose line, green = 95% isodose line, blue = 90% isodose line).

Median time to start radiotherapy following surgery was 29 days (range, 4–107 days). Surgical cavity volume was contoured as a CTV using CT simulation image registered with post-operative MRI. The median volume of the post-operative cavity in all patients was 15 cm^3^ (range, 3.8–93.3 cm^3^). Patients were treated with radiotherapy to a median dose of 40.05 Gy/15 daily fractions over 3 weeks at 2.67 Gy per fraction (range, 30–40.05 Gy in 10–15 fractions) (Figure 1). The majority (51/56) were treated to 40.05 Gy/15 fractions.

## Systemic Therapy

Thirty-nine of 56 (70%) received systemic anti-neoplastic therapy, with 22 starting treatment prior to their course of IFRT and 17 starting systemic therapy following completion of IFRT. None received systemic anti-neoplastic therapy concurrently with IFRT.

## Local Recurrence

**Figure 2:**
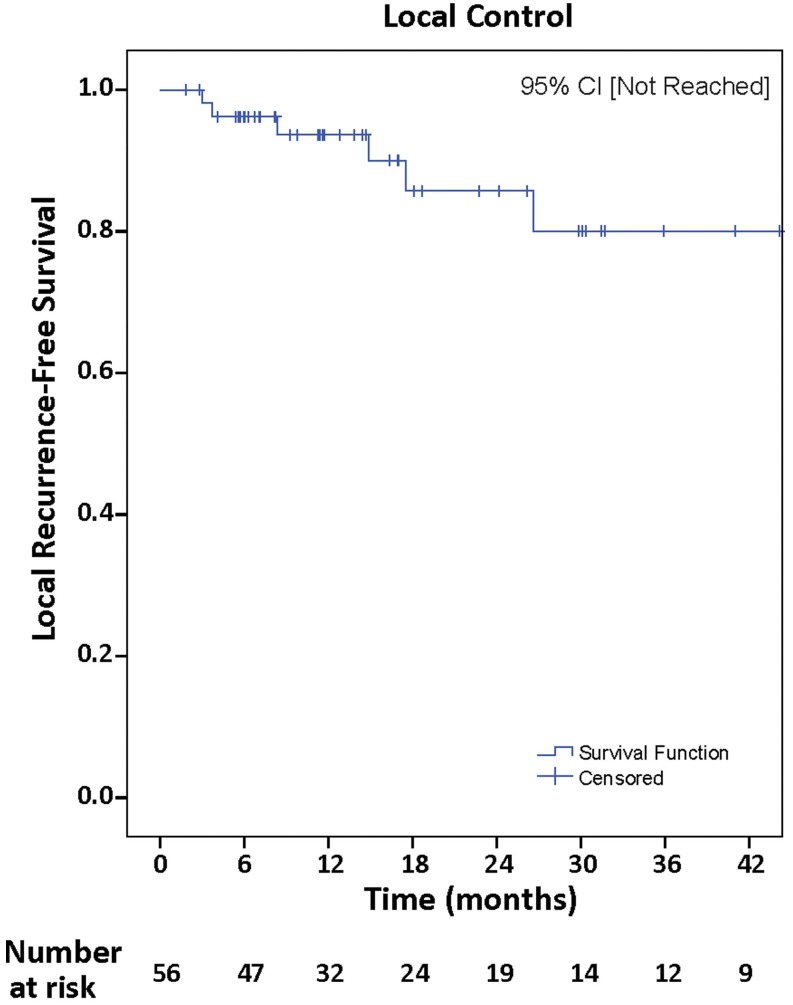
**Kaplan–Meier analysis of local recurrence-free survival at the surgical bed**.

Median follow-up was 19.1 months (range, 1.8–93.9 months), with local control achieved in 48 (85.7%). Actuarial rates of LR-free survival at 12 and 24 months were 91.4 and 85.1%, respectively, 95% CI [not reached] (Figure 2). Univariate Cox regression analysis demonstrated no clinical variables associated with increased incidence of LR including, NSCLC histopathology (*p* = 0.52), tumor maximal diameter ≥3 cm (*p* = 0.78), piecemeal resection of tumor (*p* = 0.41), and superficial tumor depth with involvement of dura mater (*p* = 0.73).

## Distant Failure

**Figure 3:**
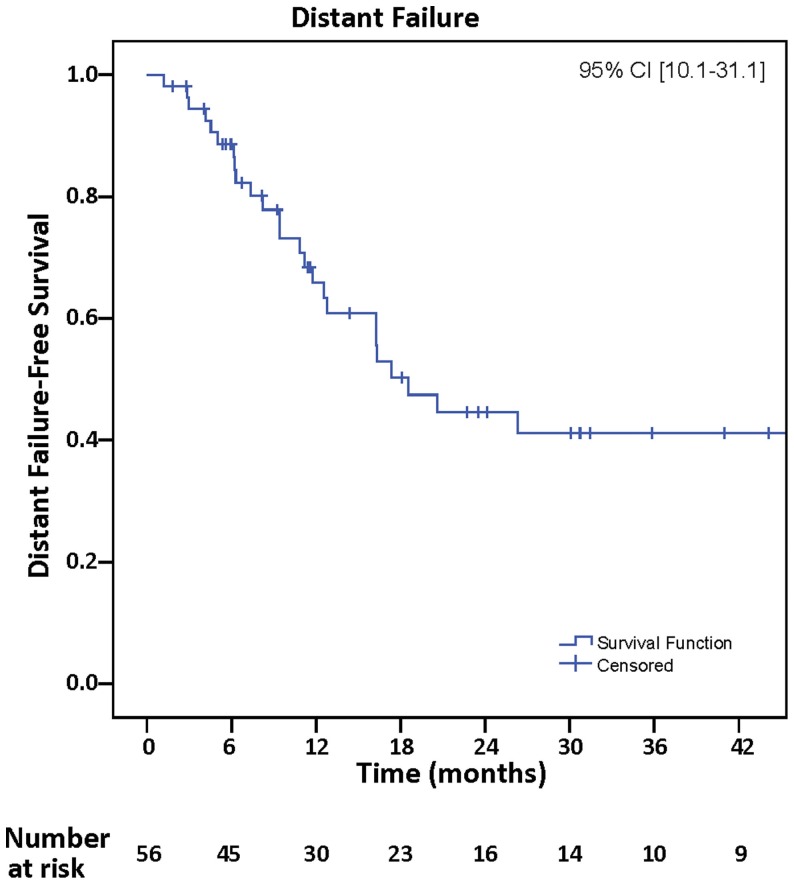
**Kaplan–Meier analysis of distant failure-free survival in the CNS**.

Distant failure was observed in 29 (51.8%), and actuarial rates of DF-free survival at 12 and 24 months were 68.4 and 45.7%, respectively, 95% CI (10.1–31.1) (Figure 3). Univariate analyses of all clinical variables demonstrated that melanoma histopathology [*p* = 0.03, hazard ratio (HR) = 2.46, 95% CI (1.06–5.74)] and infratentorial location [*p* = 0.02, HR = 2.51, 95% CI (1.15–5.49)] were associated with increased incidence of DF. Melanoma histopathology [*p* = 0.02, HR = 2.70, 95% CI (1.16–6.28)] and infratentorial location [*p* = 0.03, HR = 2.70, 95% CI (1.23–5.91)] continued to be associated with DF on multivariate analyses. Thirteen of 29 (45%) who developed DF had infratentorial tumors, and 5/10 who presented with leptomeningeal spread had infratentorial tumors.

## Leptomeningeal Spread

Following IFRT, 10/56 (18%) developed leptomeningeal spread. Five had single BM located in the posterior fossa, and three had BM superficial in location. Histopathology was not significantly associated with increased risk of leptomeningeal spread.

## Intracranial Patterns of Failure

Patterns of intracranial failure were recorded, with 3 (5%) having LR, 21 (38%) having DF, and 5 (9%) having both LR and DF.

## Overall Survival

At most recent follow-up, 69.1% of patients were alive. Survival was not significantly associated with RPA class. Median survival was 19.1 months (range, 2–94 months). Twelve and 24-month OS rates were 77.7 and 61.1%, respectively (figure not shown). Of all patients who died, three died of neurologic death from progression of intracranial disease, seven died of death due to progression of extracranial disease, one died of natural cause unrelated to malignancy, and six died of unknown cause.

## Salvage Treatment

LR were salvaged in four patients – two with surgery and SRS, one with SRS, and one with surgery and WBRT. DF were salvaged in 24 patients – 16 patients with SRS, three with WBRT, two with a second course of IFRT, two with chemotherapy and IFRT, and one with chemotherapy alone.

## Complications of Treatment

Two presented with ≥grade 2 adverse effects of treatment with CNS necrosis [CTCAE Version 4.0]. One patient with diagnosis of BRAF mutated metastatic melanoma, developed symptomatic radiation effect with headaches 2 years following her course of IFRT to 40.05 Gy/15 fractions as seen on brain MRI, with extensive confluent FLAIR and T2-weighted hyperintensity, but no evidence of disease progression in the area of prior radiation treatment. Her symptoms are presently being managed with steroids, at a dose of 40 mg daily. Another patient with NSCLC, initially treated with IFRT to 40.05 Gy/15 fractions, developed histologically confirmed radionecrosis that was initially thought to be a LR on surgical resection following salvage treatment of distant BM with SRS. Late neurocognitive effects of treatment were not systematically recorded in the medical record.

## Discussion

We report the only series using IFRT following surgical resection of single BM in 56 patients with a median follow-up of 19.1 months, expanded from our previous series of 33 patients (23). A 12-month local control rate of 91.4% is consistent with results reported by the Patchell study (90%), and compares favorably with other series of focal treatments with 12-month local control rates ranging from 74 to 94% (18–22, 24–30) (Table 2). The 12-month DF rate in our series was 32.6%, which compares favorably with other series ranging from 28 to 69% (18–22, 24, 25, 27–30) (Table 2). These favorable results may be due to improvements in anti-neoplastic therapy in addition to good performance status and well-controlled systemic disease. However, with continued follow-up, 51.8% of patients developed DF.

**Table 2:** **Selected series outcomes of different treatment options following surgical resection of brain metastases**.

Author	*N*	Gross total resection (%)	Adjuvant treatment	Median dose Gy (range)	Median follow-up, months (range)	Local control at 12 months (%)	Local control at 24 months (%)	DisDistant relapse at 12 months (%)	Median survival, months (range)	Radionecrosis/adverse radiation effect (%)
Brennan et al. (18)	39	92	SRS	18 (15–22)	12 (1–94)	85	^a^	44	15 (1–94)	18
Choi et al. (25)	112	90	SRS	20 (12–30)	11 (1–87)	91	88	54	17 (2–114)	7
Do et al. (29)	30	^a^	SRS/SRT	(15–27.5)	^a^	82	^a^	69	^a^	7
Karlovits et al. (19)	52	92	SRS	15 (8–18)	13	92	^a^	44	15	0
Kelly et al. (27)	18	94	SRS	18 (15–18)	13 (3–22)	89	^a^	35	Not reached	0
Mathieu et al. (22)	40	80	SRS	16 (11–20)	13 (2–56)	74	^a^	54	13 (2–56)	5
Petr et al. (20)	72	100	I-125 seeds	^b^	16 (3–34)	93	^a^	32	14	0
Quigley et al. (21)	32	94	SRS	14 (10–18)	14	94	^a^	28	16	0
Robbins et al. (28)	85	68	SRS	16 (12–20)	11 (1–93)	81	76	58	12	8
Soltys et al. (24)	72	85	SRS	19 (15–30)	8 (0.1–81)	79	^a^	47	15 (1–81)	10
Minniti et al. (31)	101	72	SRS^c^	27	16 (6–44)	93	84	50	17	9
Ahmed et al. (32)	65	97	SRS^c^	(20–30)	9 (1–29)	87	70	51	10 (1–30)	2
Ling et al. (30)	100	81	SRS^c^	22 (10–28)	12 (0.6–87)	72	^a^	64	13	9
Patchell et al. (10)	23	100	WBRT	36 (12 fractions)	18 (17–49)	80^d^	^a^	^a^	10	^a^
Patchell et al. (12)	49	100	WBRT	50.4 (28 fractions)	12	90^d^	^a^	14^d^	12	^a^
Patchell et al. (12)	46	100	None		11	54^d^	^a^	37^d^	11	^a^
Postop IFRT study	56	100	IFRT	40.05 (30–40.05)	19 (2–94)	91	85	33	19 (2–94)	4

*^a^Not reported*.

*^b^Activity in mCi, not Gy*.

*^c^Multi-session SRS*.

*^d^Actuarial incidence with no report at 12 or 24 months*.

With comparable rates of local control, SRS and brachytherapy have advantages compared to IFRT with respect to treatment duration. In our series, IFRT was delivered predominantly over 3 weeks, in contrast to SRS and brachytherapy, which can be delivered in a single day. The disadvantages of brachytherapy include risk of infection, limited availability, and operator experience (31). The primary disadvantage of SRS is difficulty of target volume identification following collapse of the surgical cavity. Additionally, removal of larger tumors (≥3 cm) may leave a surgical cavity too large for safe delivery of single-fraction SRS (18, 22, 24, 25, 29). The primary arguments in favor of IFRT include ease of defining PTV, reproducible treatment planning, and treatment of tumor resection sites too large for single-fraction SRS. Alternatively, multi-dose SRS has been proposed as a treatment option for patients with large tumor resection cavities (≥3 cm); however, this may come at a cost of either decreased local control or increased risk of adverse treatment effects. In a series of 101 patients with a median surgical cavity volume of 17.5 cm^3^, patients were treated with a multi-dose SRS regimen of 27 Gy in three fractions, resulting in a 9% risk of radionecrosis or adverse radiation effect. In another series with 65 patients and a median surgical cavity volume of 8.1 cm^3^, treatment with multi-dose stereotactic radiotherapy (SRT) resulted in a local control rate of 70% at 24 months (32, 33). The median surgical cavity volume in our series using IFRT was 15 cm^3^, with a local control rate of 85% at 24 months, and resulting in low events of radionecrosis or adverse radiation effect (4%). Patients presenting with larger surgical cavity volumes will, therefore, be selected for treatment with IFRT instead of multi-dose SRT not only to avoid increased risk of toxicity from treating larger volume surgical cavities but also due to physician preference.

Previously identified risk factors including larger size, involvement of meninges, and surgical resection technique (piecemeal versus *en bloc*) did not adversely affect local control in our series on univariate analysis (34, 35). This is in contrast to the results of a phase II study where tumor size ≥3 cm and superficial tumors with dural/pial involvement were associated with increased LR in patients treated with SRS following surgery (18). These discrepancies may be due to larger PTV margins (10 mm) used in this study compared to the margins (2–3 mm) used in the phase II study.

On multivariate analysis, infratentorial location and melanoma histopathology were associated with DF. These risk factors are consistent with other series using SRS following surgical resection of BM (18, 36). The increased risk of DF in patients with infratentorial BM may be due its location proximal to the cerebellar cisterns, leading to increased seeding of the cerebrospinal fluid (37). An association between melanoma histopathology and DF was supported by a series of patients treated with surgical resection followed by SRS (25). Despite this association with increased DF, it is unclear what is the appropriate therapeutic choice for this specific population due to the introduction of immunotherapy and targeted therapy (38, 39).

Over half of the patients in our series developed DF with continued follow-up. This highlights the importance of salvage treatment options to address LR and DF. We have utilized a variety of salvage approaches tailored to specific clinical situations, including WBRT with or without exclusion of the prior IFRT volume as well as SRS alone.

Although treatment was well tolerated with minimal acute and late toxicity, one important limitation of this study is the absence of neurocognitive testing. Another limitation of this study is the number of patients evaluated in this study, the heterogeneity of dose regimens used in this study (30–40.05 Gy/10–15 fractions), the lack of information regarding targetable mutations or rearrangements to further characterize this selected population, and the inherent biases associated with a retrospective study. Yet, our motivation for our treatment approach is to decrease the risk of neurocognitive toxicity as one randomized trial demonstrated measurable decline at 4 months with SRS followed by WBRT compared to SRS alone using the Hopkins Verbal Learning Test-Revised (13, 15). Due to the solid theoretical and retrospective evidence that minimizing treatment of normal brain is a worthwhile clinical goal, it will be difficult to directly compare IFRT with WBRT in a randomized trial for patients with surgically resected single BM. However, an interesting question is to evaluate the effectiveness and toxicity of SRS versus IFRT in this patient population. Another worthwhile approach may be consideration of a tighter margins using IFRT to further spare normal brain.

In conclusion, we demonstrate feasibility and safety of using IFRT following surgical resection of single BM for patients with good performance status and well-controlled primary disease. IFRT can be considered as an alternative therapeutic approach to SRS for patients with resected metastases and surgical resection cavities ≥3cm, and in lieu of WBRT for these select patients with close follow-up.

## Author Contributions

All authors (SS, RV, MT, AN, JG, DK, and JS) have contributed to the conception, draft/revision, approval of final draft, and are accountable for all aspects of work in ensuring that questions related to accuracy or integrity of work are appropriately investigated and resolved.

## Conflict of Interest Statement

The authors declare that the research was conducted in the absence of any commercial or financial relationships that could be construed as a potential conflict of interest.
